# Automated single particle detection and tracking for large microscopy datasets

**DOI:** 10.1098/rsos.160225

**Published:** 2016-05-18

**Authors:** Rhodri S. Wilson, Lei Yang, Alison Dun, Annya M. Smyth, Rory R. Duncan, Colin Rickman, Weiping Lu

**Affiliations:** 1 Institute of Biological Chemistry, Biophysics and Bioengineering, Heriot-Watt University, Edinburgh EH14 4AS, UK; 2 Edinburgh Super-Resolution Imaging Consortium, www.esric.org; 3 OmniVision Technologies, Co., Ltd, 4275 Burton Drive, Santa Clara, CA 95054, USA

**Keywords:** particle detection, tracking, microscopy, photoactivated localization microscopy

## Abstract

Recent advances in optical microscopy have enabled the acquisition of very large datasets from living cells with unprecedented spatial and temporal resolutions. Our ability to process these datasets now plays an essential role in order to understand many biological processes. In this paper, we present an automated particle detection algorithm capable of operating in low signal-to-noise fluorescence microscopy environments and handling large datasets. When combined with our particle linking framework, it can provide hitherto intractable quantitative measurements describing the dynamics of large cohorts of cellular components from organelles to single molecules. We begin with validating the performance of our method on synthetic image data, and then extend the validation to include experiment images with ground truth. Finally, we apply the algorithm to two single-particle-tracking photo-activated localization microscopy biological datasets, acquired from living primary cells with very high temporal rates. Our analysis of the dynamics of very large cohorts of 10 000 s of membrane-associated protein molecules show that they behave as if caged in nanodomains. We show that the robustness and efficiency of our method provides a tool for the examination of single-molecule behaviour with unprecedented spatial detail and high acquisition rates.

## 1. Background

Particle tracking is an indispensable tool in the analysis of time-lapse microscopy datasets. It is usually modelled as a correspondence problem, in which particles are first detected in all images before being linked from frame to frame. For a tracking method to perform well, the particles must first be detected and localized with the highest possible accuracy before they are linked to form particle trajectories. However, in live-cell microscopy, there is always a compromise between image quality and cell viability due to effects such as photo-bleaching and photo-toxicity. This together with the need for high frame-rate acquisition results in images of a low signal-to-noise ratio (SNR). Particle detection typically follows the structure of denoising and signal enhancement before segmentation through simple thresholding or more complex methods such as Bayesian segmentation [1]. However, correct detection becomes increasingly more difficult as SNR deteriorates. Typically, both denoising and signal enhancement are required to achieve a reliable result, though the former operation is sometimes avoided when the task is to localize particles rather than the recovery of the image [2–6]. Moreover, deconvolution can also be used for reducing blur in images to achieve better results [6,7]. There are now dozens of software tools available for particle tracking, most of which follow the traditional deterministic approach of particle detection before trajectory linking [2,8–12], though another class of probabilistic particle tracking methods is becoming more popular [13,14]. In comparison, probabilistic methods, such as multiple hypothesis tracking (MHT) [15,16] and Sequential Monte Carlo (SMC) methods [17], employ spatio-temporal filtering to estimate particle location and, in general, outperform deterministic methods. However, probabilistic methods require more prior knowledge about the dynamics and organization (i.e. cluttering and density) of particles; multi-frame filtering is also computationally expensive [18]. A recent publication based on the ‘International Symposium on Biomedical Imaging (ISBI) Particle Tracking Challenge’ [19] presented a comprehensive comparison of 14 tracking methods, including many cited above. The authors have also made available the database of the synthetically generated images with ground truth to others in the field as an excellent resource for further tests. The results from the challenge can be considered as the benchmark for further development in the field.

In this paper, we present an automated particle detection and tracking algorithm for large fluorescence microscopy datasets in low-SNR environments. The algorithm builds on our previous detection framework in which signal enhancement is achieved by exploring the concept of the particle probability image (PPI), which is a non-local mapping of the original greyscale image through the use of multi-scale Haar-like features [20]. In this paper, we further develop and improve this framework in all areas. Firstly, the particles are detected through soft thresholding of the PPI, which enables it to work more robustly in low-contrast and low-SNR environments. Secondly, the particles are detected without sophisticated image denoising, which not only makes it more efficient, but also avoids image over-smoothing and, therefore, the loss of small and weak particles [2–6]. Thirdly, the trajectory linking model has been extended to include the morphology and intensity profiles of particles, leading to more accurate particle linking, particularly in dense particle fields. Finally, the algorithm has now been automated, requiring five simple parameters as an input that can be estimated from image observations and are iteratively refined for optimal detection. This is important to make it more accessible to biologist users. We begin by comparing the present and previous algorithms, and show a significant improvement in detection, localization and tracking. We next validate the algorithm using the synthetic ‘vesicles’ and ‘receptors’ scenarios from the ISBI database and then extend to include a real single vesicle (large dense-cored vesicles (LDCVs)) dataset with a manually identified ground truth. We further corroborate and test it by generating new real single-molecule datasets, acquired from distinct cell types (primary central neurons and neuroendocrine cells) with both high sensitivity using an electron multiplying charge coupled device (EMCCD) detector and a state-of-the-art high-speed complementary metal-oxide semiconductor (sCMOS) detector, with a range of temporal sampling rates of up to 100 frames per second (fps) and under noisy imaging conditions where the SNR was approaching 1. To the best of our knowledge, tracking of 10 000 s of single molecules with a high temporal sampling rate and low-SNR conditions has yet to be achieved. This work would open the way for cell biology to analyse the dynamics of particles in large datasets for statistical appraisal.

## 2. Material and methods

The key to our particle detection algorithm lies in the concept of the PPI which was first introduced in [21], and was shown to significantly enhance particle features in low-SNR and low-contrast environments. The PPI is formed using non-local and statistic mapping by using Haar-like features, which measure local contrast in different sizes as well as shapes, providing a better response than Laplacian of Gaussian [22] for detecting particles of different geometries. Subsequent particle linking is developed over the interacting multiple model (IMM) filter [2,9,15,16], in which particle motion modelling and data association have been improved by incorporating extra information about the morphology and intensity profiles of particles from the detection algorithm; this leads to a greater accuracy in multiple particle tracking. The method is described below as a two-part process of particle detection followed by particle linking.

### 2.1. Particle detection

Particle detection comprises six steps. The algorithm is now automated, requiring five parameters from the user that can be estimated from image observations. These parameters are iteratively refined for optimal detection. The input parameters of the algorithm are: typical particle size (*S*) and minimum particle size (*s*), both are area in the unit of pixel number, expected number of particles per image (*N*), the particle density and the image SNR. The estimated particle density and image SNR are adaptively changed so that the number of detected particles approaches the expected number of particles per image (see below).

**Figure 1.**
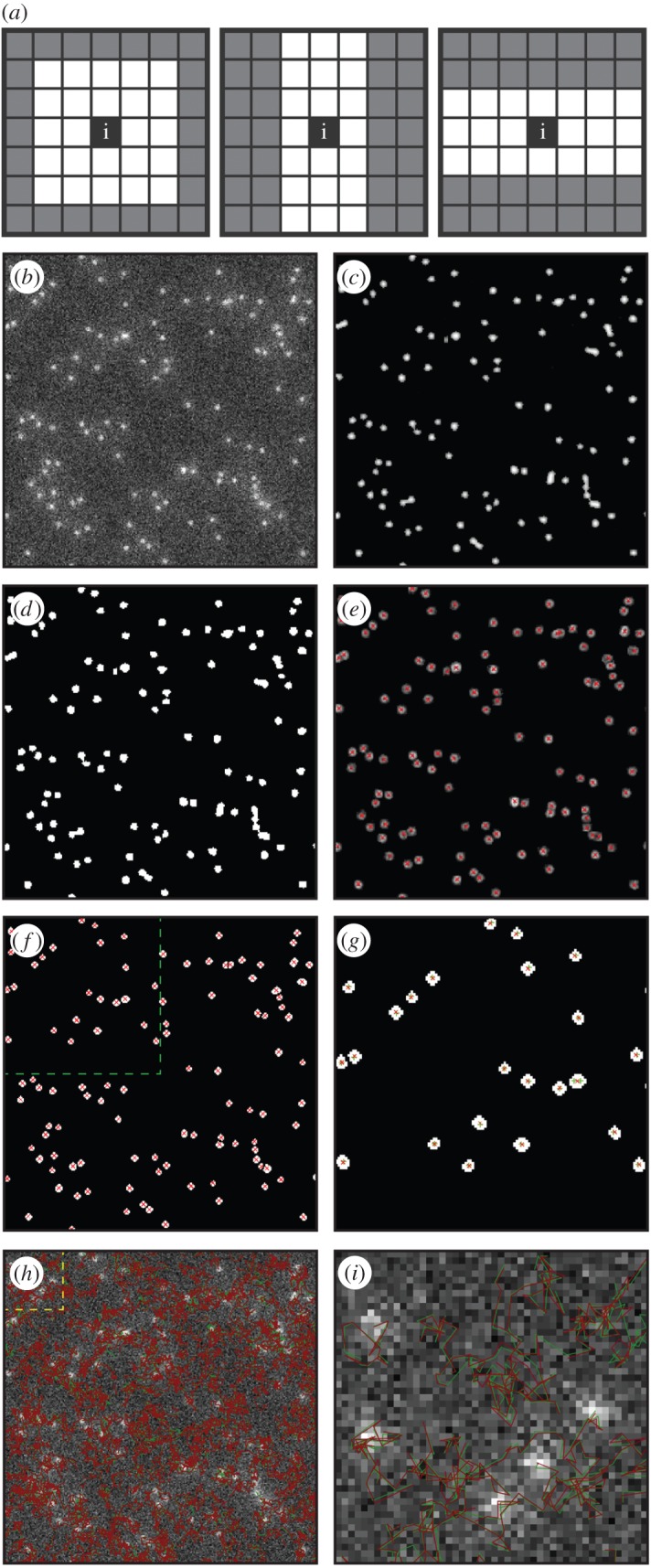
The method for particle detection and trajectory linking. (*a*) Three Haar-like features used to calculate local contrast. (*b*) Synthetic vesicle image with SNR = 4 and medium density from the ISBI database (top-left quarter only). (*c*) PPI. (*d*) PERs. (*e*) Gradient image with markers for watershed. (*f*) Particle segmentation results where red crosses mark refined particle locations. (*g*) Comparison of particle localizations between results (red crosses) and ground truth (green crosses) in the zoom region marked in (*f*). (*h*) All found particle tracks (red) and ground truth tracks (green). (*i*) A zoom region of (*h*).

(1) *PPI construction*. The PPI is constructed to enhance particle features, especially, in low-SNR and low-contrast environments, so particles can be robustly identified even when they are barely visible in a raw (greyscale) image. To construct the PPI from a raw image (figure 1*b*), we first calculate the Haar-like features [21], namely, the local contrast for each pixel between a small (white) area centred at the pixel and its immediate (shaded) surrounding. The features are obtained by convolving the exemplar kernels in figure 1*a*, at different spatial scales with the original greyscale image. The maximum value from each spatial scale at each pixel is then combined to form a Haar feature image (contrast image). Every pixel in the Haar feature image is then classified into the class of particle or background according to their strength; a pixel with a higher feature value is statistically more likely to belong to a particle. The parameters (*S* and *N*) stated above are used along with a particle enhancement factor (*F*), determined by the SNR and particle density of the image, to set an appropriate threshold for this classification. Particle probability in each pixel is finally calculated from the resultant binary image, and is set as the ratio of the number of particle pixels to the total number of pixels in an area of the typical particle size centred at that pixel (figure 1*c*).
(2) *Particle existing regions* (*PERs*). PERs are initially estimated in the PPI by simply setting an appropriate threshold of PPI≥1/e  because of their single-peak distribution. PERs are then refined for each individual region based on the relative strength of particle probabilities within the PPI (figure 1*d*). Compared with the previous method, the PERs are now obtained through soft thresholding that depends on the values in the PPI in each of the local areas.
(3) *Particle (foreground) markers*. Particle markers correspond to local maxima in the raw image and must have a PPI value greater than half the maximum PPI value within the corresponding PER. For numeric stability, the local maxima are obtained in a noise-reduced image, which has been obtained by convolving the raw image with a Gaussian kernel of the standard deviation$\;\sqrt{s}$. We note that by restricting markers within PERs, we remove an overwhelming number of false markers, making the simple local maximum localization robust in low-SNR environments.
(4) *Marker-controlled watershed segmentation*. Segmentation using the watershed transform algorithm does not work well in low-SNR environments, so markers are introduced to improve the performance; this method is referred to as marker-controlled watershed segmentation [23]. We apply the particle markers found in step 3 to the gradient image of the raw data, adding our PER identified in step 2 as a further restriction (figure 1*e*) for image segmentation; the result of the watershed segmentation is shown in figure 1*f*. We note that by restricting the particle boundary to within a PER, the particle boundary becomes more accurate; this restriction becomes important when the gradient is no longer robust enough to determine the particle boundary, i.e. in the presence of strong noise. Particle segmentation is therefore refined by incorporating PERs from the PPI and watershed in the raw image.
(5) *Particle localization*. We define a particle's localization as the intensity-weighted centre-of-mass in terms of greyscale intensity within the PER. The localizations for all the detected particles are shown by crosses in figure 1*f*. To compare the result with the ground truth, we show the top-left area of figure 1*f* in figure 1*g*, where good overlapping between our localizations (red crosses) and ground truth localizations (green crosses) can be observed. A missed detection involves only two spatially very close particles, the distance between the two is about two pixels, similar to the dimension of minimum particle size, $\sqrt{s}$, that we defined in the beginning. We note that, in general, intensity-weighted centre-of-mass gives a more robust result of particle localization than local intensity maximum in low-SNR conditions. However, when typical particle sizes are very small, say, less than nine pixels, the particle shape may not be estimated accurately through segmentation, and local intensity maxima by using 2D Gaussian fitting is a better way to determine the final particle localization in this case [11].
(6) *Adaptive parameter setting*. the parameters are adaptively refined in an iterative manner; given the five initially estimated parameters, we compare the number of detection results with the number of estimated particles, *N*, and if the difference between the two values is not within an acceptable range, the SNR and/or particle density are re-evaluated using a search methodology until the difference is in the accepted range. We also note that for image sequences, where the number of particles varies noticeably from frame to frame, the initial value of the estimated particle number is updated as the number of particles detected in the previous frame.

### 2.2. Particle linking

The linking program is also automated, requiring the input of two basic parameters of ‘maximum linking distance’ (in pixels), which relates to the maximum velocity of a particle, and ‘minimum number of frames’ for a trajectory (typically three frames).

Trajectory linking is developed over the IMM filter [2,9,15,16], which is made up of multiple Kalman filters and is capable of switching between filters to best suit the previous motion of the particle. Particle motion is considered to be one of three models: random walk, first- and second-order linear extrapolation relating to Brownian motion, constant speed and constant acceleration, respectively. The IMM filters follow the three well-established steps of state predication, data association and filtering parameter update, as described in the earlier work [20]. Here, we have improved the state prediction by extending the state space variables to include the morphological features, such as area, circularity and major/minor axis lengths, as well as the intensity profiles of particles, enabling more accurate trajectory linking in dense particle fields. Figure 1*h* shows all detected (red) and ground truth (green) tracks. For comparison, a small area of the top-left corner of figure 1*h* is magnified in figure 1*i*, and this shows good overlapping between the two sets of tracks.

### 2.3. Analysis of performance improvement

**Figure 2.**
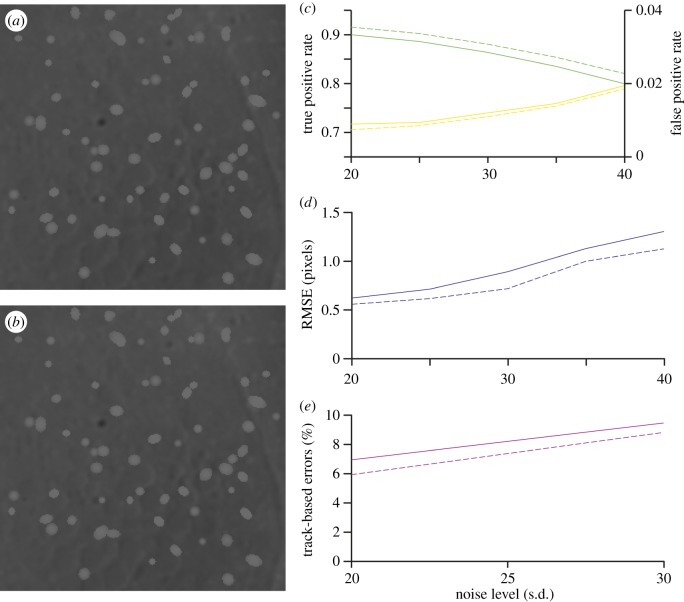
Quantification of improvements. (*a*,*b*) Noise-free image and corresponding noisy image as used in [20]. The latter has been corrupted with added Poisson noise and Gaussian noise with mean *m* = 5 and s.d. *σ* = 30 for 8-bit data. (*c*) Comparison of true positive rate (green) and false positive rate (yellow) of our current (dashed line) and previous (solid line) particle detection algorithm for increasing noise levels. (*d*) RMSE of true positive detections for our current (dashed line) and previous (solid line) methods. (*e*) Track-based errors (equation (2.1)) for our current (dashed line) and previous (solid line) particle tracking method.

To quantify the improvements of our current method over the previous method, we compare them directly using the same synthetic data (figure 2*a*,*b*) and metrics as presented in [20]. First, we examine the detection accuracy of the algorithms using the true positive rate (TPR) and false positive rate (FPR). As seen from figure 2*c*, the current detection algorithm has resulted in a consistent increase of the TPR (green lines) by 20% (2% in absolute terms) while also decreasing the already extremely low FPR (yellow lines) for all noise levels. This improvement can be attributed to the more robust way that the particle markers are identified in the present method. Next, we calculate localization accuracy of both algorithms using the root mean squared error (RMSE) of true positive detections. Figure 2*d* shows an improvement of between 10 and 20% in the RMSE, and the improvement increases as the noise increases in the images. This result can be attributed to our new thresholding scheme for generating the PER that uses all information within the image, compared with the previous method that only used foreground (particle) information, allowing for more accurate segmentation and therefore a more accurate centre-of-mass calculation for localization. Finally, we calculate the track-based errors [2], which are defined as

$$E_{\mathrm{track}}^{*}=1-\left(\frac{T_{\mathrm{correct}}^{*}}{T_{\mathrm{total}}}\right),$$

where $T_{\mathrm{total}}$ is the total number of ground truth tracks; $T_{\mathrm{correct}}^{*}=\sum_{i=1}^{T_{\mathrm{total}}}\left(Y_{\mathrm{tracked},i}/Y_{i}\right)$ is the number of correctly computed trajectories, which is computed as the sum of the ratio of correctly tracked time steps $Y_{\mathrm{tracked},i}$ to the total time steps $Y_{i}$ for all the tracks, similar to its definition in [2]. We again see a consistent improvement of about 15% (1% in absolute terms) in the track-based errors (figure 2*e*) for all noise levels. These improvements in track accuracy are in line with the improvement to the detection rates identified in figure 2*c*.

### 2.4. Experimental procedure

All fluorescence microscopy data were acquired using an Olympus Cell Excellence Total Internal Reflection Fluorescence (TIRF) microscope with a 150× 1.45 NA objective with cells maintained in 5% CO_2_ and 37°C throughout. For vesicle tracking data, the images were acquired on an EMCCD detector (Hamamatsu) at 20 Hz under TIRF illumination with a 491 nm laser. For single particle tracking photoactivated localization microscopy (sptPALM) imaging, molecules were cyclically activated (405 nm laser) and excited (561 nm laser) under TIRF illumination. All molecules were bleached before the next cycle of activation. These experiments were performed with an EMCCD (Hamamatsu) detector for the primary central neurons and an sCMOS (Hamamatsu) detector for the neuroendocrine cells.

### 2.5. Software

The software for running the tracking method is included in the electronic supplementary materials accompanying this paper. The software is currently implemented in Matlab (Mathworks, Cambridge) with the image processing toolbox and is run through a single line command in the command window, it will run faster with a parallel processing toolbox, although this is not essential. The software was provided as executable p-code and the user interface of the software provides step-by-step guidance for users to run the software. The software can be used to reproduce all the results in the manuscript and users are able to import their datasets and produce their own results for evaluation. The run times stated in this manuscript are for a standard laptop computer running Microsoft Windows 8 with 16 GB of memory and a 2.4 GHz Intel i5 processor using the parallel processing toolbox within Matlab with 4 workers. We note that implementation of our algorithm in higher level programming languages, such as C or C++ will result in a better computational performance. However, we have focused this paper primarily on the efficiency of the method, so it will run comparatively efficiently in other implementations.

## 3. Results and discussion

### 3.1. Validation of method using synthetic data

**Figure 3.**
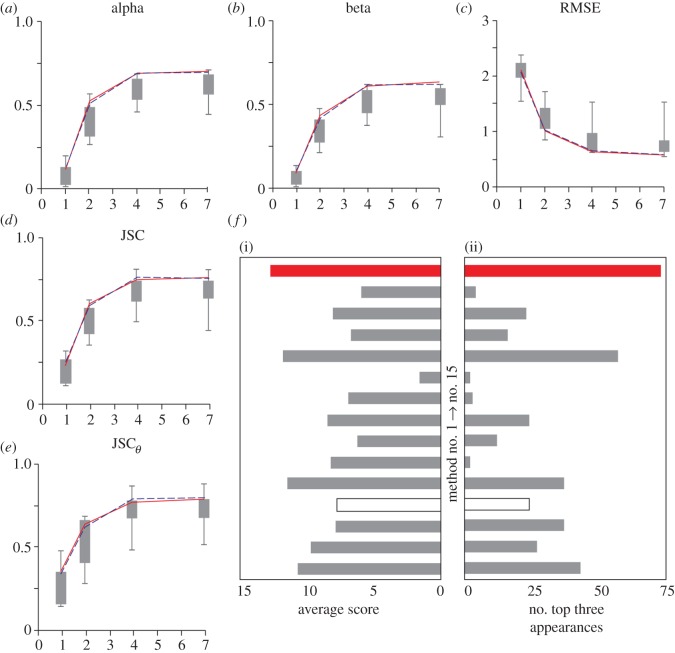
Performance comparison on synthetic data. (*a*–*e*) Comparison of our method (training data denote solid red line; test data, dashed blue line) with the 14 methods (grey box plots) on the five metrics for the synthetic vesicles (scenario 1) at medium particle density. (*f*(i)) Number of top three appearances for each of the 15 methods for the vesicles and receptors at different levels of particle density and noise. (i) Average score of all 15 methods. Note that in (*f*) method 4 is represented by an empty bar as it only completed the challenge on scenario 2 which is not investigated here.

We first tested our tracking method on image scenarios 1 and 3 of the ISBI database [19], corresponding to synthetic vesicles and receptors at different noise levels and particle densities in (2*D* + *t*) space.^1^ A representative result for the ‘vesicles’ at medium density by our tracking method, denoted as method 15 (training data denote solid red curve; test data, dashed blue curve), is given in figure 3*a*–*e*, along with those by all other 14 methods (represented as a grey box plot, where the lines stretch from the lowest to the highest values of the 14 measurements) taken from the data supplied with the original publication. Our method resides consistently among the top performers for all five metrics at all SNR levels. It in fact works equally well for low particle densities of the vesicle scenario, as well as the receptor scenario. We note that while the performance for high density is not as good, this is due to the linking framework not being designed for high-density data. Table 1 shows the top three performers for the two synthetic scenarios investigated at different SNR and density levels. We plot the number of top three appearances for each method in figure 3*f* (right), as this was used to determine the best method in the ISBI challenge [19]; our method has 73 out of a possible 120 top three appearances—15 more than the next ranked method (method 11). To quantify the performance of the 15 methods in a different way, we assigned a simple point scheme; 15 points to the number 1 performer for any given metric on any given dataset, 14 points to the number 2 performer and so on, giving ranks to all of the methods. Quantification in this way is fairer for methods that rank lower overall as it rewards methods that perform consistently instead of methods that achieve a few top three appearances. The top three ranked here are still the same as the top three ranked as in [19], albeit one order change. As shown in figure 3*f* (left), the top three methods, with our method (15) now included, when scored in this way are 15, 11 and 5 scoring on average 12.7, 11.7 and 11.4 points, respectively, over the 24 datasets and five metrics. The complete results for our method in comparison with the other 14 methods on all synthetic-vesicle and -receptor datasets can be found in the electronic supplementary material, figures S1 and S2, respectively. We note that the processing time of the method varies with the particle density and SNR of the dataset being investigated. For the low-density datasets with 500 tracks, the runtime varied from 21 to 72 s with the runtime increasing as the SNR decreases. For the medium-density datasets with 2500 tracks, the runtime varied from 85 to 185 s. Finally, for high-density datasets with 5000 tracks, the runtime varied from 191 to 384 s. Furthermore, the ground truth for test datasets was not available at the time of our early investigation, so we undertook the work on the training datasets, after a conversation with one of the lead authors in the challenge confirmed that the training and test data produce little difference (I Smal 2015, personal communication). In the light of the recent availability of the ground truth for the test data, we have undertaken validation on the mid-density vesicles to show, indeed, the measurement is almost identical (figure 3*a*–*e*).

**Table 1.** Top three performers. The top three performers for each of the five metrics (five rows) at three levels of particle density (low, middle and high) and four levels of noise (SNR = 1, 2, 4, 7), for the datasets of scenarios 1 (vesicles) and 3 (receptors) in [19]. The numbers within the table correspond to the competing methods in [19] with our method added as method 15.

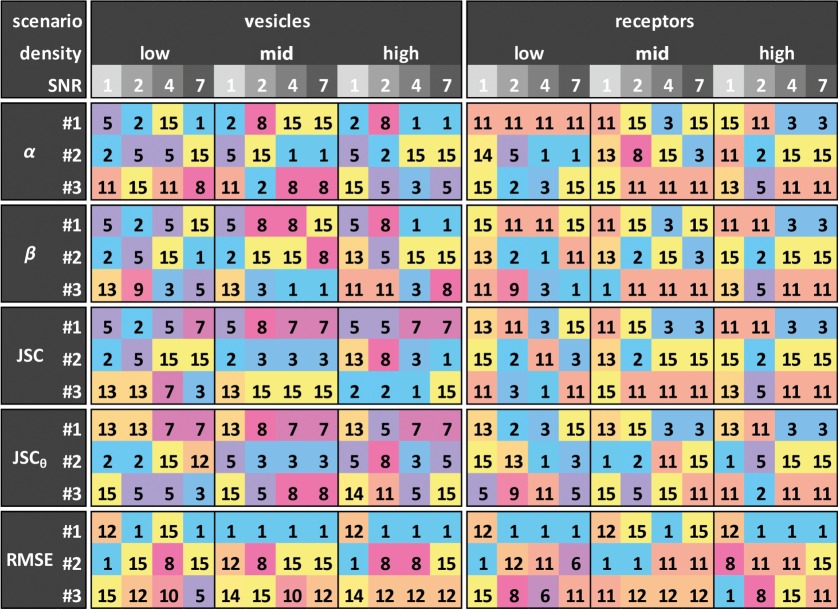

### 3.2. Validation of method using experimental data

**Figure 4.**
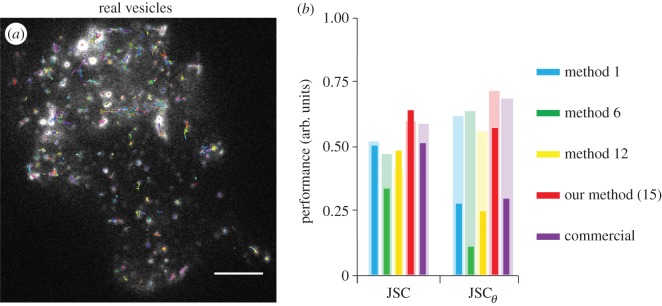
Performance comparison on experimental data. (*a*) The first of 250 image frames of single LDCVs of PC12 cells, acquired under TIRF illumination at 20 fps, with 267 × 275 pixels and scale bar: 5 µm. All the tracks laid on the image have a minimum tracked length of three frames. (*b*) Performance comparison against the JSC and $\mathrm{JS}C_{\theta }$ metrics, blue for method 1, green for method 6, yellow for method 12, red for our method (15) and purple for Imaris; the faded bars correspond to their performance on the synthetic vesicles at a similar level of noise and particle density.

Even well-modelled synthetic datasets, such as those provided by the ISBI challenge, are unlikely to mimic real-world data accurately. To validate our approach using real data, we first tracked single EGFP-labelled LDCVs in rat phaeochromocytoma (PC12) cells, acquired under TIRF illumination at 20 fps using an EMCCD detector. Figure 4*a* shows the first image of a sequence of 250 frames with all the tracks recorded by our method from the whole dataset overlayed (in arbitrary colours for contrast). These images have more complex spatial and temporal features compared with those in synthetic datasets: larger fluctuations of peak intensities, uneven background and intensity-dependent noise. The SNR, measured using the same method as in [19], of the image sequence was estimated to be on average 3.2. However, owing to fluctuation of intensities over the image, the SNR was measured for many manually identified single vesicles throughout the image sequence, with background being defined as a region adjacent to the vesicle, and the value 3.2 was obtained from an average of around 200 such vesicles. The particle density was found to represent the ‘medium’ level of the ISBI database. We manually identified all vesicles in each frame and subsequently linked them to form ground truth tracks for comparison. The results of tracking by our method, along with three other exemplar methods [8,11,24,25] given as methods 1, 6 and 12 in the ISBI challenge and also the commercial software Imaris (Bitplane), are given in figure 4*b*. Methods 1, 6 and 12 were chosen as their software was readily available for use and they represent a high, medium and low performing method from [19]. For analysis, we only use the JSC and $\mathrm{JS}C_{\theta }$ metrics used in the ISBI challenge as the metrics *α*, *β* and RMSE measure the accuracy of tracks through the distance between ground truth and measured tracks, as the localization accuracy of manually identified tracks can be unreliable and the resultant values of the metrics would also be unreliable. We find that our method gives higher values for JSC and $\mathrm{JS}C_{\theta }$ compared with the others, indicating a higher specificity and sensitivity in its ability to detect individual vesicles in each frame and more correct tracks in the image sequence, respectively. It is also interesting to compare these results with those for the synthetic-vesicle data of the ISBI database, which are displayed as the faded bars in figure 4*b* and act as the benchmark for the metrics on our real vesicle data. As seen, our method shows a good consistent performance compared with the benchmark, despite its more complex behaviours, while the other four methods perform notably less well in comparison. The runtime for our software on this exemplar dataset with around 1500 tracks was 202 s, which is consistent with the runtimes from the synthetic data previously examined.

### 3.3. Biological applications

**Figure 5.**
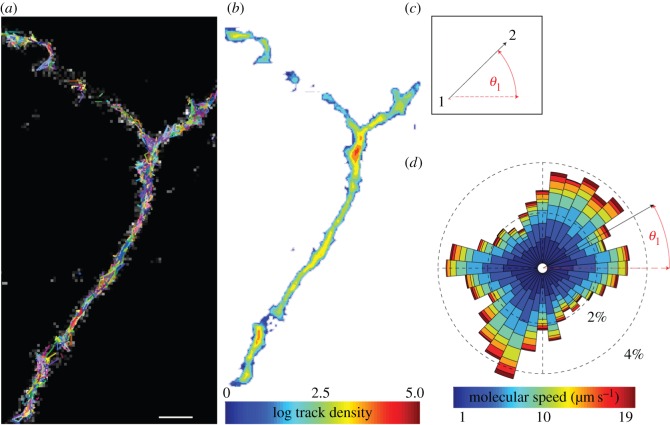
Tracking of real single molecules at 30 fps. (*a*) A typical image (171 × 256 pixels) containing large cohorts of single Munc18-1 molecules overlaid with tracks from an image sequence of 2000 frames acquired at 30 fps, scale bar, 1 µm. (*b*) Track density image, the background is in white, showing the number of tracks passing through each region of the image. (*c*) The angle measuring the direction of motion *θ*_1_. (*d*) Rose diagram showing the collective dynamics of the direction of motion, the dominant directions of motion align with axon orientation.

A major goal of modern biology is to be able to track large cohorts of single molecules; this presents significant challenges as individual fluorophores can emit a low number of photons before photo-destruction and high frame rates are required to avoid under-sampling, thus noise dominates these datasets. To address these hurdles, we applied our particle tracking method to two distinct real datasets, each presenting their own limitations and challenges, using photoactivatable monomeric-Cherry-labelled Munc18-1 molecules (a protein required for neurotransmission [26]) acquired using a sptPALM protocol as previously described [27]. First, an image sequence of 2000 frames was acquired on an EMCCD detector at 30 fps from live primary cortical neuron cultures. The SNR of these images was measured to be about two and due to the nature of PALM imaging the particle density could be assumed to be in the low--mid range. Particles were detected in all frames using our new particle detection algorithm, and a little over 4000 tracks subsequently formed using our linking framework with the total processing time of the dataset being around 15 min, which is consistent with the times previously mentioned for the synthetic ISBI challenge data. Figure 5*a* displays all identified trajectories (in arbitrary colour for contrast) laid on the first image of the sequence (in grey), whereas, figure 5*b* shows the corresponding density of trajectories, namely, the number of tracks cumulated per unit area in this image sequence. To understand the dynamics of individual molecules and their collective behaviour, we measure the direction (angle) of motion (*θ*_1_) for each inter-step of all the trajectories and display them as ‘rose diagrams’, as depicted in figure 5*c*. Rose diagrams are essentially angular histograms, where the colour and angle of a ‘petal’ section describe the distance and angle a particle moves between two consecutive frames, and the size of each petal section describes the number of movements at that angle and length. Molecules move along axons in neurons, manifesting as the strong off-centre vertical line on the rose diagram in figure 5*d* that is aligned with the axon orientation. When the molecules accumulate in varicosities, the movement is essentially Brownian, which can be seen as an inner circle in figure 5*d*, the slight horizontal extensions from this inner circle are due to particle movement along a secondary part of the axon as seen in the top left of figure 5*a*.

**Figure 6.**
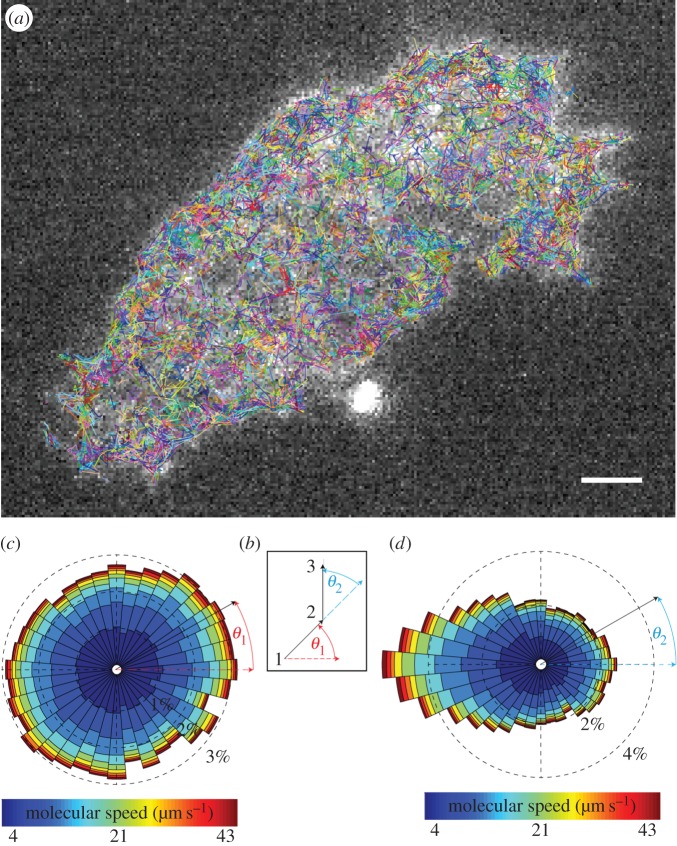
Tracking of real single molecules at 100 fps. (*a*) A typical image (258 × 198 pixels) of SNAP-25 molecules in neuroendocrine cells acquired under TIRF illumination at 100 fps, scale bar, 1 µm; 202 890 tracks (shown coloured) are obtained from the dataset of 180 000 frames and overlaid on the first image of the sequence. (*b*) The angles measuring the direction of motion, *θ*_1_, and direction switching, *θ*_2_. (*c*) Rose diagram showing the collective dynamics of direction of motion. (*d*) Rose diagram showing the collective switching in direction of molecules.

Appropriate high-speed data acquisition, certainly higher than rates that can be achieved using highly sensitive EMCCD detectors, is required to avoid sampling artefacts in dynamic biological imaging of fast processes, such as molecular diffusion [28]. We therefore extended our work to high-speed imaging of single molecules in neuroendocrine cells using labelled SNAP-25 (the most abundant pre-synaptic protein, also essential for regulated hormone secretion [26]), taking advantage of the large surface area of these cells compared with neurons. The images were acquired under TIRF illumination at 100 fps for 30 s per PALM cycle with a total of 60 cycles acquired resulting in a 180 000 frame dataset, using a sCMOS detector. Owing to the high frame rate and lower sensitivity of this detector, the image quality is poor; SNR was measured on average as 1.45. However, our method can robustly identify the molecules in individual frames at this noise level, the detection accuracy was confirmed by manual checks at different time points throughout the dataset. Again, the particle density is low--mid and hence favourable for trajectory linking, the subsequent trajectories we checked for accuracy before analysis. A dataset of this size took approximately 6 h to process and analyse. A total of more than 200 000 tracks were found, and these can be seen overlaid in figure 6*a*. Because of the large surface area of these cells, we measured two angles for each inter-step of a trajectory: the direction of motion (*θ*_1_) as well as direction switching (*θ*_2_), as depicted in figure 6*b*. The molecules were found to have no collective preference on the direction of motion in figure 6*c*. However, by inspecting the inter-step angle *θ*_2_, we have found that if a molecule initially travels along one direction, it is more likely to switch to the opposite direction in the next step, so showing collectively a strong asymmetry in direction switching in favour of $\theta_{2}\approx 180^{\circ }$ (figure 6*d*). These molecules thus appear to move as if caged in nanodomains, an interesting observation that may agree with earlier hypotheses around membrane organization of palmitoylated proteins [29–31].

## 4. Conclusion

In this paper, we have developed and improved our previous work [20] on single-particle tracking and applied it to very large and challenging fluorescence biological datasets. The further developments in our previous work allow us to more reliably identify particles, particularly in low-SNR environments, allowing for more accurate linking in dense particle fields and efficient computation of very large datasets. The implementation of an interactive interface for the algorithm, along with a reduced number of parameters that can be derived from observations, means that our automated method can be operated with little prior experience. Furthermore, adaptive parameter setting means that the input parameters can be refined even if the initial estimates are not accurate. Application to the synthetic database showed that our method performed among the best of the current state-of-the-art methods in terms of the five commonly used metrics. It performs especially well on images of low-SNR with low or mid particle density and so is particularly suited to the main application in this paper to sptPALM data, which has the characteristics of low density and low-SNR. In the absence of sophisticated denoising, our method is able to process hundreds of thousands of images and particle trajectories on a desktop computer. We have further shown that it can handle more complex imaging environments, such as inhomogeneity of intensity and intensity-dependent noise. Finally, our analysis supports the previous observation about the caged effect on the organization of palmitoylated proteins at the cell membrane [29–31].

## Supplementary Material

Supplementary Figures - Full results from testing on ISBI Challenge Data

## Supplementary Material

Supplementary Software - Matlab Implementation of the Tracking Software

## Supplementary Material

Supplementary Dataset - Image dataset used for figure 4

## Footnotes

1 Scenarios 2 (microtubules) and 4 (viruses) were not considered for validation due to the incompatibility of their localization (asymmetric Gaussian peak) and dimensionality (three-dimensional image data), respectively, with our tracking method.

## Data accessibility

The ISBI challenge data are available from their website at http://bioimageanalysis.org/track/. We have provided the image data for figure 4 in the electronic supplementary materials. The datasets for figures 5 and 6 are too large to be uploaded here as electronic supplementary materials, but the authors will provide these datasets upon request. The software was provided as executable p-code, the user interface of the software provides step-by-step guidance for users to run the software. They can run the software to reproduce all the results in the manuscript and are able to import their datasets and produce their own results for evaluation. We are currently unable to provide the source code at this stage, as part of the algorithm is being used for ultrasound imaging applications, which is being considered for a patent application for medical imaging and diagnosis. The supporting data are available on Dryad: http://dx.doi.org/10.5061/dryad.6kg29.
