# Supplementary material for: Automated single particle detection and tracking for large microscopy datasets
Source: R Soc Open Sci. 2016 May 18;3(5):160225. doi: 10.1098/rsos.160225 (PMC4892463; doi:10.1098/rsos.160225)
Supplement: Supplementary Figures - Full results from testing on ISBI Challenge Data [file rsos160225supp1.pdf]

Automated Single Particle Detection and Tracking for Large Microscopy Datasets.

Rhodri S. Wilson[1,2], Lei Yang[3], Alison Dun[1,2], Annya M. Smyth[1,2, ‡], Rory R. Duncan[1,2], Colin Rickman[1,2], and Weiping Lu[1,2]

[1]*Institute of Biological Chemistry, Biophysics and Bioengineering, Heriot-Watt University, Edinburgh EH14 4AS, UK*

[2]*Edinburgh Super-Resolution Imaging Consortium, www.esric.org*

[3]*OmniVision Technologies, Co., Ltd, 4275 Burton Drive, Santa Clara, CA 95054.*

[‡]*Present address: Research Governance & QA Office, University of Edinburgh, The Queen's Medical Research Institute, 47 Little France Crescent, Edinburgh, EH16 4TJ.*

Supporting Information

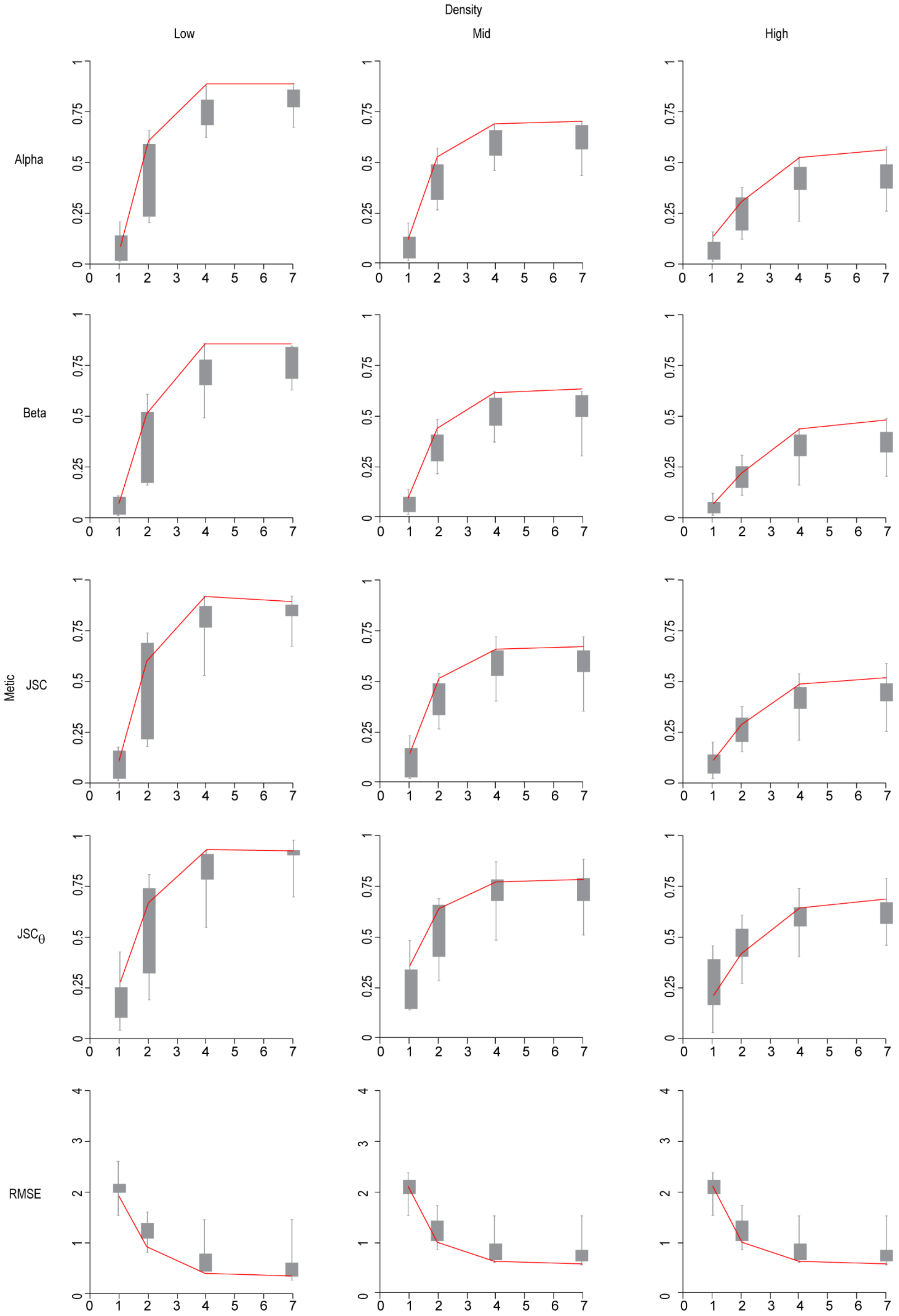

Density
Low
Mid
High
Alpha
Beta
Metic
JSC
JSC$_\theta$
RMSE

S1 Fig. Synthetic Vesicle Performance. Performance comparison between our method (red) and the 14 methods in [19] (gray) for the synthetic vesicle data at 4 different SNR levels and three particle densities, using the five metrics. These results are incorporated in Table 1.

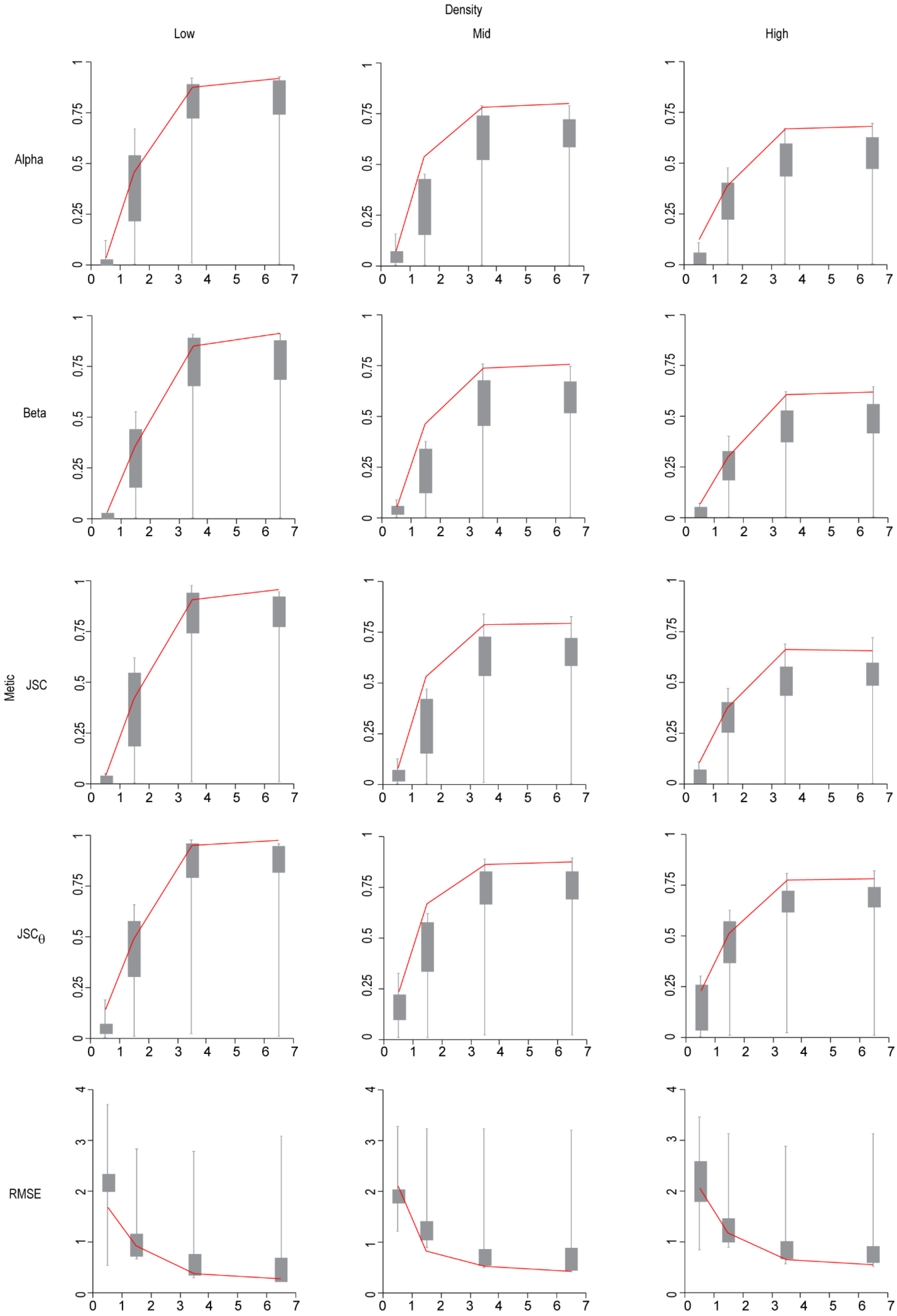

Density
Low
Mid
High
Alpha
Beta
Metic
JSC
JSCθ
RMSE

S2 Fig. Synthetic Receptor Performance. Performance comparison between our method (red) and the 14 methods in [19] (gray) for the synthetic receptor data at 4 different SNR levels and three particle densities, using the five metrics. These results are incorporated in Table 1.

S3 Software. Particle Tracking Method implemented in Matlab. Code for running the particle detection algorithm and linking framework in Matlab. A software guide is also included to help the user through the software.
